# SPOT-Cardio: Integrated Application for AI-Powered Automated Myocardial Scar Quantification on Joint Bright- and Black-Blood Late Gadolinium Enhancement MRI Images

**DOI:** 10.3390/jcm14238428

**Published:** 2025-11-27

**Authors:** Kun He, Edouard Gerbaud, Thaïs Génisson, Victor de Villedon de Naide, Théo Richard, Kalvin Narceau, Mathilde Merle, Maxime Sermesant, Matthias Stuber, Hubert Cochet, Aurélien Bustin

**Affiliations:** 1 IHU LIRYC, Heart Rhythm Disease Institute, Hôpital Xavier Arnozan, Université de Bordeaux–INSERM U1045, Avenue du Haut Lévêque, 33604 Pessac, France; kun.he@ihu-liryc.fr (K.H.); edouard.gerbaud@chu-bordeaux.fr (E.G.); thais.genisson@ihu-liryc.fr (T.G.); victor.de-villedon@ihu-liryc.fr (V.d.V.d.N.); theo.richard@ihu-liryc.fr (T.R.); kalvin.narceau@ihu-liryc.fr (K.N.); mathilde.merle@ihu-liryc.fr (M.M.); maxime.sermesant@inria.fr (M.S.); mstuber.mri@gmail.com (M.S.); hcochet@wanadoo.fr (H.C.); 2 Department of Cardiovascular Imaging, Hôpital Cardiologique du Haut-Lévêque, CHU de Bordeaux, Avenue de Magellan, 33604 Pessac, France; 3 Cardiology Intensive Care Unit and Interventional Cardiology, Hôpital Cardiologique du Haut-Lévêque, CHU de Bordeaux, Avenue de Magellan, 33604 Pessac, France; 4 Centre Inria d’Université Côte d’Azur, 06902 Sophia Antipolis, France; 5 Department of Diagnostic and Interventional Radiology, Lausanne University Hospital and University of Lausanne, Rue du Bugnon 46, 1011 Lausanne, Switzerland; 6 Center for Biomedical Imaging (CIBM), 1015 Lausanne, Switzerland

**Keywords:** cardiovascular disease, cardiac MRI, artificial intelligence, medical imaging software, image segmentation, myocardial scar, automated diagnosis

## Abstract

**Background/Objectives:** Cardiac magnetic resonance (CMR) imaging is a key tool for diagnosing cardiovascular disease, but its analysis remains time-consuming and dependent on expert interpretation, which can limit throughput and reproducibility. To address these challenges, we aim to develop an automated solution that streamlines CMR post-processing, enabling consistent, rapid, and quantitative assessment of cardiac structures and myocardial pathology. **Methods:** We introduce SPOT-Cardio, an AI-powered imaging analysis toolbox based on a 2D breath-held late gadolinium enhancement (LGE) imaging technology: SPOT. This acquisition combines BR- and BL-LGE images in a single scan, allowing simultaneous capture of high-contrast scar information and detailed cardiac anatomy. Using the resulting CMR images, deep learning models (based on 2D U-Net or MedFormer) were trained to segment cardiac structures and myocardial scars. The trained models and associated image-processing algorithms were then integrated into the open-source medInria platform and specifically within its cardiac-focused MUSICardio application. **Results:** SPOT-Cardio enables automatic segmentation of cardiac structures and myocardial scars, performs landmark-based regional localization, and extracts key biomarkers such as scar volume, extent, and transmurality. The resulting quantitative measures are presented in standardized bullseye plots accompanied by detailed clinical reports. **Conclusions:** With a one-click workflow and intuitive visualization, SPOT-Cardio reduces manual workload and supports more accessible and consistent cardiovascular assessment. By integrating advanced image acquisition with AI-based automation, it provides a practical and efficient solution for streamlined and quantitative CMR analysis.

## 1. Introduction

Cardiovascular disease (CVD) remains the leading cause of death in Europe, affecting millions annually and posing a major challenge to global health. Early detection and accurate diagnosis are critical to reducing mortality, as emphasized by the World Health Organization’s goal to cut CVD-related deaths by 25% by 2025 [1,2].

When blood flow is restricted, typically due to coronary artery disease, parts of the myocardium (the muscular tissue of the heart) can become deprived of oxygen and causing cardiomyocyte death. In response, the body forms myocardial scars, composed of fibrous tissue that replaces dead cells. These scars are most often found in the left ventricle (LV), are a key marker of prior heart injury. Their detection and characterization are essential for understanding heart disease and guiding treatment strategies.

Modern non-invasive imaging techniques allow visualization and assessment of internal structures and functions without the need for surgical intervention or penetration of the body. In cardiology, common non-invasive modalities include cardiac magnetic resonance (CMR) image, computed tomography (CT), echocardiography, and nuclear imaging (Single Photon Emission Computed Tomography/ Positron Emission Tomography), which enable evaluation of myocardial structure, function, perfusion, and fibrosis with minimal risk to the patient [3]. Among these, CMR stands out as the gold standard for comprehensive, radiation-free assessment of cardiac function and tissue characterization. It is particularly powerful for detecting and quantifying myocardial edema and scar, which are clinically important because these different patterns are observed in several cardiovascular diseases, including acute coronary syndromes, chronic structural heart disease, and ventricular arrhythmias. Accordingly, CMR plays a critical role in the diagnosis, risk assessment, and management of a wide range of cardiovascular diseases.

Late gadolinium enhancement (LGE) techniques, especially bright-blood (BR-LGE) sequences using an inversion recovery mechanism, are cornerstone techniques in CMR. BR-LGE sequences enhance the contrast between healthy and damaged myocardium, which helps in both diagnosis and prognosis [4,5]. Recent studies have indicated that the spatial distribution of myocardial scars may carry important prognostic significance, as more heterogeneous or dispersed scars can disrupt myocardial electrical conduction, thereby increasing the risk of ventricular arrhythmias and adverse cardiac events [6]. However, the bright signal from the blood pool often reduces contrast between myocardial lesions and adjacent blood, making subendocardial scars difficult to detect, and making it difficult to quantify scar extent, to locate the scar, and to identify the transmurality [3,7]. To overcome this limitation, black-blood (BL-LGE) imaging was developed, offering improved visualization of myocardial scars, but it remains limited by insufficient anatomical delineation [8,9,10]. More recently, combined BR- and BL-LGE in a single acquisition have emerged, allowing for the simultaneous capture of high-contrast scar information (from BL-LGE images) and detailed anatomical structure (from BR-LGE images). This significantly improves scar detection accuracy and clinical utility, thus improving diagnostic confidence and facilitating artificial intelligence (AI)-based analysis. In clinical practice, myocardial scar assessment on LGE MRI can be performed using qualitative, semi-quantitative, or quantitative approaches. Qualitative methods rely on visual inspection by experienced readers, for example, identifying the approximate location and extent of hyperenhancement on LGE images, which is quick but subjective and dependent on operator experience. Semi-quantitative methods, such as the 17-segment scoring system, introduce some standardization but are still affected by inter-observer variability. Quantitative methods use signal intensity thresholds (e.g., 2SD (Standard Deviation) or 5SD above normal myocardium) or automated segmentation techniques, including threshold-based or deep learning approaches, to objectively measure scar size, providing higher reproducibility and precision. However, CMR acquisition is complex and time-consuming, often requiring extensive manual post-processing and expertise, which limits its widespread clinical use.

Meanwhile, with the sharp rise in AI, medical diagnostics are increasingly integrating automation. AI-driven tools help streamline workflows, reducing reliance on time and specialized expertise, thereby improving diagnostic efficiency and consistency. In cardiovascular imaging, AI models have been successfully applied to automatically segment cardiac structures, quantify myocardial function, and detect pathological features in echocardiography, cardiac CT, reducing manual workload while improving reproducibility [11,12]. Similarly, AI-driven analysis of electrocardiograms enables early detection of arrhythmias and other cardiac abnormalities, supporting timely clinical intervention [13]. Moreover, AI tools are being investigated for wider clinical applications in cardiology and electrophysiology, such as patient risk stratification, management of care, and integration with real-time decision support systems [14]. CMR in particular requires AI to overcome the challenges of traditional analysis, including time-consuming procedures, manual annotations, and limited reproducibility. Recent AI developments also integrate BR- and BL-LGE MRI techniques to enable efficient, automated detection and quantification of myocardial scar [15].

Despite these advancements, a significant clinical gap persists: there is still no dedicated and user-friendly analysis tool that allows radiologists to fully exploit the diagnostic potential of joint BR- and BL-LGE imaging data, regardless of AI integration.

To address this need, we propose SPOT-Cardio: a novel AI-powered imaging analysis software based on the SPOT (Scar-specific imaging with Preserved myOcardial visualizaTion) imaging technology [16], which simultaneously captures myocardial scar characteristics and anatomical information data. SPOT-Cardio integrates AI to enable fast, automated, and accurate segmentation and quantification of cardiac structures and myocardial scars. Developed within the MUSICardio [17] application built on the open-source medical imaging platform medInria [18], this solution supports efficient, user-friendly workflows with one-click operation, to drastically reduce manual intervention.

The main pipeline focuses on:

- AI-driven segmentation of cardiac structures (LV wall)
- Automated myocardial scar detection and quantification (based on the LV contours)
- One-click operation for rapid and precise result generation

By automatically extracting key cardiac biomarkers such as scar volume, extent, and transmurality, SPOT-Cardio improves diagnostic accuracy and simplifies the analysis process, making cardiovascular disease management more efficient and easier to implement.

## 2. Architecture

### 2.1. Components

**Figure 1:**
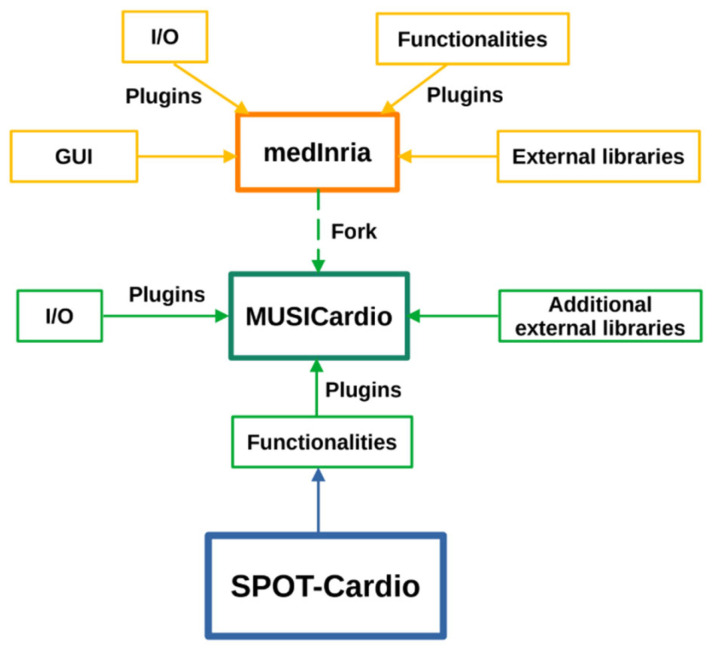
An overview of the integration of the SPOT-Cardio module within the medInria and MUSICardio platforms.

SPOT-Cardio is one of the functional workspaces integrated into MUSICardio, which is a platform aiming to provide a wide range of functionalities and processing pipelines dedicated to cardiology applications for both diagnosis and therapy guidance. It is built on the open-source software medInria v4.0, which includes support for import/export formats, different functional plugins, and external libraries. It includes a comprehensive set of tools for image segmentation, visualization, filtering, histogram analysis, data reformatting, registration, and mesh processing. All these tools are provided to the community within a user-friendly common framework for enhanced efficiency. The modular plugin system of medInria allows the seamless integration of additional features specifically tailored for cardiac applications, making MUSICardio v4.1 a flexible and extensible tool for cardiac imaging analysis. Figure 1 illustrates the architecture of the MUSICardio application, which is built around its medInria core. SPOT-Cardio v1.0 is integrated into this architecture as a dedicated plugin for cardiac scar and anatomy analysis.

### 2.2. Libraries

The application core is programmed in C++, and its interface is implemented using the Qt library (https://www.qt.io (accessed on 18 November 2025)). The image processing components of the entire toolbox are implemented using the ITK library v5.0.0 [19] and other additional external libraries that are already integrated into the MUSICardio application plugins.

In our C++ development workspace, where AI capabilities are deeply integrated, we selected ONNX Runtime [20] as our machine learning inference engine after evaluating available options. The library provides efficient cross-platform support for models trained in major frameworks, including PyTorch, TensorFlow, and scikit-learn, through the standardized ONNX format. Our evaluation showed that ONNX Runtime delivers optimized performance across diverse hardware configurations, from CPUs to GPUs and specialized accelerators. Key deciding factors include its comprehensive documentation and significantly lower deployment complexity compared to alternatives like TensorFlow’s C++ API, making it particularly suitable for our workspace requirements.

The main external libraries used are summarized in Table 1.

## 3. Algorithm

This workspace is developed based on the AI-powered SPOT imaging framework that builds upon the recently proposed 2D SPOT sequence [25,26,27,28,29,30]. The SPOT technique enables simultaneous acquisition of co-registered BR- and BL-LGE images, providing superior myocardial scar assessment compared to conventional methods. By exploiting AI, the framework can automatically extract ventricular contours from BR-LGE images (anatomical information) and propagate them to BL-LGE images (scar information) for precise scar identification. In the following sections, we will briefly introduce these two key technological components.

### 3.1. SPOT Image Acquisition

**Figure 2:**
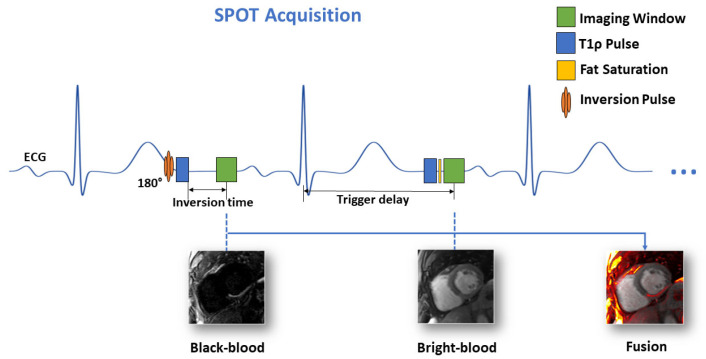
Schematic of the proposed joint BR- and BL-LGE SPOT sequence.

The SPOT technique represents an advanced CMR approach that simultaneously acquires co-registered BR- and BL-LGE images during a single breath-hold examination [16,25]. SPOT is an ECG-triggered single-shot based on balanced steady-state free precession (bSSFP) sequence for image acquisition, using an alternating contrast preparation scheme: during odd cardiac cycles, a 180° non-selective inversion pulse combined with T1-rho (T1ρ) preparation module generates optimal BL-LGE contrast for enhanced scar detection, while even cardiac cycles utilize T1ρ preparation alone to maintain BR-LGE contrast for anatomical reference and LV localization (Figure 2). Each slice requires ten cardiac cycles, including two initial “dummy” heartbeats for magnetization stabilization, followed by eight acquisitions to capture four images of each contrast type at mid-diastole. The comprehensive protocol acquires from 10 to 20 short-axis slices through multiple breath-holds to achieve full ventricular coverage, using optimized parameters including 1.5 × 1.5 mm^2^ in-plane resolution, 8 mm slice thickness, 500 Hz spin-lock frequency with 27 ms duration (for the T1ρ module), 60° flip angle, and parallel imaging acceleration (GRAPPA factor 2, 36 reference lines). The reconstruction pipeline performs four signal averages and generates real-time fused color-coded displays combining both contrast mechanisms, with processing completed within 10 s for efficient clinical workflow.

### 3.2. AI-Powered SPOT Image Processing

**Figure 3:**
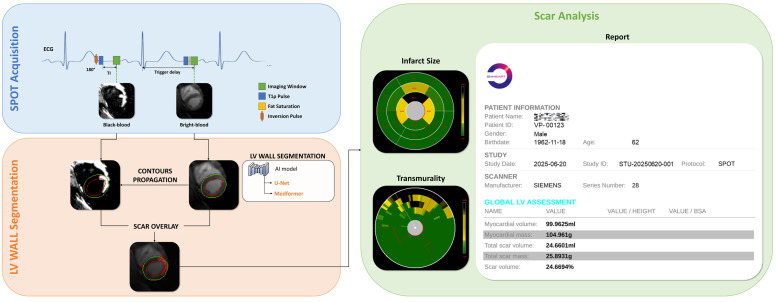
Framework for fully automated scar detection and quantification from joint BR- and BL-LGE SPOT images, with the corresponding analysis report for an anonymous patient, including a metric summary.

The AI-powered image processing methodology (Figure 3) is based on an optimized 2D U-Net or Medformer deep learning model, trained on an extensive dataset comprising over 4300 BR-LGE SPOT images from 288 patients with structural heart disease. The model architecture, refined through a binary cross-entropy loss function and ADAM optimization with carefully selected parameters (learning rate = 10^−4^, batch size = 32, epochs = 200), achieved good precision in automatic LV wall segmentation. The obtained contours are then propagated to the co-registered BL-LGE SPOT images, where scar tissue is automatically identified using thresholding methods (e.g., Otsu [26]), AI-based detection and region growing [31] techniques, as described in subsequent sections. The system then automatically computes key scar characteristics, including scar mass, scar extent, and scar transmurality. Validation in 12 independent test cases demonstrated 89% accuracy for scar detection and 75% accuracy for transmurality assessment, showing excellent agreement with manual analysis (R^2^ = 0.93). The automated image processing time is approximately 10 s compared to 17 ± 7 min for manual processing.

## 4. Functionalities

### 4.1. SPOT Workspace

**Figure 4:**
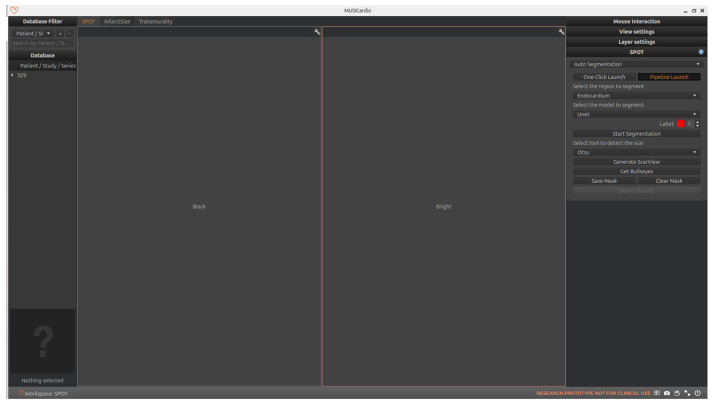
SPOT Workspace: the interface displays the patient browser (**top left**), a dual-view for synchronized visualization of co-registered BR- and BL-LGE SPOT images (**center**), and analysis toolboxes (**right**). The toolboxes include pipeline and one-click execution modes, with buttons for loading segmentations, scar detection and quantification, adjusting visualization settings, and exporting results.

As shown in Figure 4, the SPOT workspace is a core component of the MUSICardio platform, designed to facilitate the visualization and analysis of CMR images. The interface provides two synchronized image views: the left view displays BL-LGE images, while the right view displays BR-LGE images. This dual-view configuration enables clinicians to compare both image modalities simultaneously for a more comprehensive assessment.

On the right-side panel, users can access two modes for executing the image analysis pipeline: “pipeline launch” mode and “one-click launch” mode.

In the “pipeline launch” mode, the user proceeds step-by-step through three primary functions that form the core of the workspace. This cardiac image analysis system integrates automated segmentation of cardiac anatomical structures, scar tissue detection, and comprehensive scar quantification using American Heart Association (AHA) 16-segment bullseye plot analysis. Initially, the platform identifies relevant regions of interest in the LV through AI-based segmentation. Then, it detects the presence and extent of myocardial scar tissue. Finally, the system quantifies the scar by generating AHA 16-segment bullseye plots to evaluate both infarct size and transmurality.

In the “one-click launch” mode, the system enables automated execution of the entire pipeline. Before starting, users can configure settings such as the segmentation region, AI model, and scar detection method. The system then sequentially performs all processing steps.

In both modes, users have the possibility to export the results in the chosen format, which includes all computed segmentation masks and quantitative metrics. In the following sections, we detail the technical implementation of each component and also explain how users can interact with the interface to execute each function.

### 4.2. Automated LV Wall Segmentation

**Figure 5:**
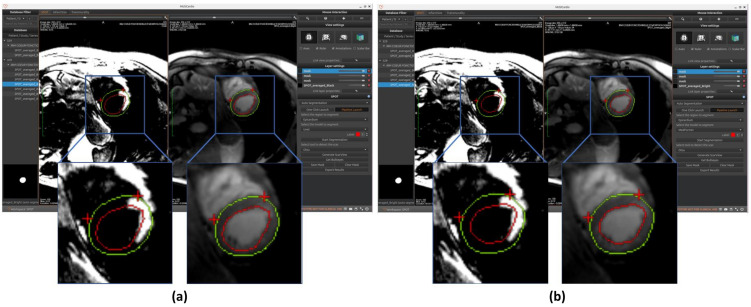
Automated segmentation of the left ventricular endocardium (red circle) and epicardium (green circle) performed on BR-LGE images using (**a**) U-Net and (**b**) MedFormer models. Contours are then propagated onto BL-LGE images allowing for scar analysis. Landmarks detection with a U-Net based model was applied in both cases on BR-LGE images to localize the right ventricular (RV) insertion point region within the myocardial muscle (two red crosses).

The first function enables automated segmentation of LV regions of interest in cardiac MRI. Within our workspace, users initially upload paired BR- and BL-LGE images, which are automatically displayed in adjacent viewing panels. The system offers three distinct segmentation targets: LV endocardium, epicardium, or full myocardial wall. Following the AI-powered SPOT framework, users may select between two deep learning architectures: a U-Net-based model or a MedFormer-based alternative. Both models are evaluated and produce correct results, as illustrated in Figure 5. Extensive testing shows that the MedFormer model demonstrates slightly better performance than the U-Net model, particularly at the basal and apical levels. Nevertheless, both models remain suitable for providing reliable references in our application. As previously detailed in Section 2.2, both models are implemented using the ONNX Runtime library for optimized inference. These models were trained on a dataset of approximately 300 patients (over 5000 images) who were referred for cardiac MRI between August 2022 and April 2023 due to known or suspected ischemic heart disease, enabling reliable automated region extraction. All participants underwent SPOT sequence imaging. Patients were excluded if they were under 18 years of age, had a history of allergic reaction to gadolinium-based contrast agents, severe renal failure, non-MR-conditional implants, inability to lie supine for 50 min, were pregnant or breastfeeding, or could not provide informed consent. All imaging was performed on a 1.5 T clinical MRI scanner (Magnetom Aera, Siemens Healthcare, Erlangen, Germany). These two model architectures will be presented in subsequent sections.

After selecting both the target region and preferred model architecture, users can also define the segmentation mask color, either by choosing from predefined options or by customizing their own. Once all parameters are configured, clicking on the “Start Segmentation” button initiates the process. The algorithm primarily operates on BR-LGE images, processing all slices simultaneously. The resulting segmentation masks are automatically mapped to the corresponding BL-LGE images for cross-reference. Typical processing time ranges from several seconds to a minute (depending on slice count), representing substantial time savings versus manual segmentation. The interface provides customizable visualization options, including adjustable mask contour colors, and allows users to either retain or discard generated segmentation masks based on clinical requirements.

Currently, after the segmentation results are generated, manual modification of the masks is not supported. However, we recognize that in certain cases, adjustments may be necessary to refine the segmentation and achieve more accurate results. To address this, future versions of the framework will incorporate the option for manual adjustments. This enhancement is intended solely to improve accuracy and precision and does not alter the overall fully automated nature of the workflow. By providing this flexibility, users will be able to fine-tune results when needed, ensuring more reliable outcomes while retaining the efficiency of automated processing.

#### 4.2.1. LV Wall Segmentation Model Based on Unet

U-Net is a Convolutional Neural Network (CNN) architecture specifically designed for biomedical image segmentation. First introduced by Ronneberger et al. [32], it is based on the Fully Convolutional Network (FCN) [33] and has been modified and extended to achieve more accurate segmentation results with fewer training images. U-Net follows an encoder–decoder structure, where the encoder (contracting path) captures contextual information, while the decoder (expanding path) enables precise localization. Additionally, it employs skip connections to transfer spatial information directly from the encoder to the decoder, improving segmentation accuracy, particularly in medical imaging tasks.

**Figure 6:**
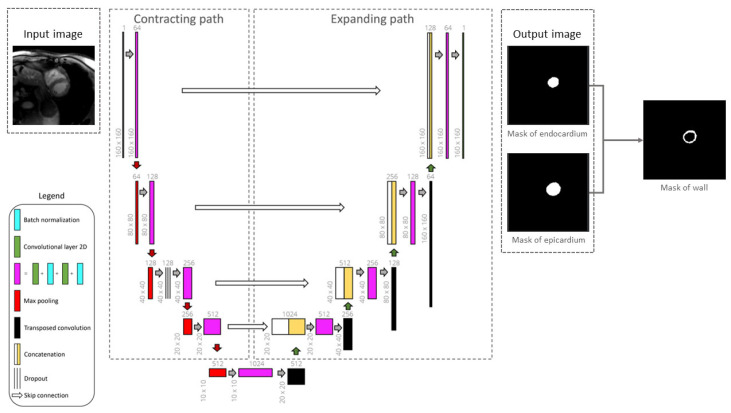
Framework of the 2D U-Net architecture for segmenting the epicardium and endocardium. Myocardial left ventricular wall is then obtained by deducting the endocardial mask from epicardial mask [15].

In this workspace, we employed a U-Net model that has been specifically optimized for cardiac MRI segmentation, as shown in Figure 6 [15]. The model has been trained using a dataset of BR-LGE SPOT DICOM images from 177 patients, with between 10 and 16 image slices for each patient. The dataset was divided into training (70%), validation (10%), and testing (20%) subsets. Before training, all images were center-cropped to 160 × 160 pixels to ensure consistent dimensions during segmentation. In addition, standard normalization was applied to scale the pixel intensity values to a range between 0 and 1. The model aimed to segment the epicardium and endocardium, with ground truth masks manually annotated by an experienced radiologist. For evaluation, a separate test set of 152 patients (unseen by the model during training) was used, each providing both BR- and BL-LGE images with 9 to 14 slices. This model achieved a Dice score of 93.0 ± 0.1% for epicardial segmentation on this test dataset, demonstrating high accuracy.

While we recognize that k-fold cross-validation or external validation could further enhance the assessment of model generalizability, the current work primarily focused on workflow integration. Further extensive model optimization and validation will be pursued in subsequent work. For this model, only the BR-LGE images are used as input, while all corresponding BL-LGE images were used for propagating the segmented contours, enabling subsequent scar analysis.

The results of segmentation of endocardium and epicardium by using the model U-Net are shown in Figure 5a.

#### 4.2.2. LV Wall Segmentation Model Based on MedFormer

MedFormer [34] is a transformer-based deep learning architecture designed for medical image segmentation tasks. Unlike traditional CNNs, MedFormer leverages the self-attention mechanism of transformers to capture long-range dependencies and global contextual information within medical images. This allows the model to better understand complex anatomical structures and variations, which is especially beneficial for segmenting organs or regions with irregular shapes and sizes.

The architecture typically integrates a hierarchical encoder–decoder framework, where the encoder extracts multi-scale features using both convolutional and transformer blocks, and the decoder reconstructs the segmentation map while incorporating global context through skip connections.

Results of endocardium and epicardium segmentation using the Medformer model are shown in Figure 5b.

### 4.3. RV/LV Insertion Points (Landmarks) Detection

Our workspace also implements an AI-based landmarks detection approach designed to localize the two right ventricular insertion points within the myocardial muscle, which is a critical step to generate both bullseyes. The pre-processing workflow begins by uniformly cropping all cardiac MRI images to a fixed resolution of 140 × 140 pixels, followed by normalization based on the dataset’s mean and standard deviation. This standardization ensures consistent input conditions for the neural network across different subjects and imaging variations.

The model used is a U-Net CNN, which produces two heatmaps as output, each corresponding to a predicted anatomical landmark. These heatmaps represent the spatial probability distribution for each target point, with values ranging between 0 and 1 across the 140 × 140 pixel grid. This allows the model to perform precise localization while capturing contextual spatial relationships. The model was trained in a supervised manner using manually annotated landmarks as ground truth references.

To further enhance robustness and mitigate the limitations of small dataset size for SPOT images, a transfer learning strategy was employed. The U-Net model was initially pre-trained on a larger dataset of short-axis cardiac cine MRI slices (*n* = 6000), spanning basal, mid-ventricular, and apical levels, with each image containing visible features relevant for insertion point identification, in order to capture general cardiac structural features. The model was then fine-tuned on the smaller SPOT dataset. This two-phase training improves convergence and accuracy when applied to more specific anatomical regions such as the insertion point.

This model, like other AI modules in our workspace, was implemented using the ONNX Runtime environment to ensure high-performance inference. Final prediction outputs are visualized on the corresponding MRI images (see Figure 5), enabling users to verify model performance through anatomical overlays.

### 4.4. Myocardial Scar Detection

The workspace’s second core capability focuses on myocardial scar detection, utilizing the unique contrast properties of SPOT sequence BL-LGE images to distinguish scar tissue from healthy myocardium. Three distinct analytical approaches are integrated: Otsu’s thresholding method provides fundamental image processing capabilities, while a specialized deep learning model offers advanced pattern recognition for scar identification. Additionally, a region-growing algorithm enables precise boundary delineation from user-defined seed points. Within the workspace, users can freely select their preferred scar detection methodology. Once selected, clicking the corresponding “Generate ScarView” button initiates the scar detection process. All scar detection operations are performed on BL-LGE images, with results automatically transferred to corresponding BR-LGE images for each cross-sectional slice. This visualization allows users to validate scar presence by comparing both modalities side by side. Similarly, as with automatic segmentation, we plan to incorporate a manual adjustment feature for scar detection results in future versions. This improvement aims to further enhance the accuracy and completeness of the detection, ensuring that the final results are more reliable and precise. The operational principles and implementation details of these complementary detection methods will be thoroughly examined in subsequent sections.

#### 4.4.1. Scar Detection with Otsu

In the BL-LGE image, scar tissue typically exhibits near-maximum pixel intensities (approaching 255) while healthy myocardium appears much darker (approaching 0). To leverage this inherent contrast difference, we first perform adaptive image pre-processing to optimize the contrast characteristics based on the specific intensity distribution of each BL-LGE image. This pre-processing step ensures robust performance across varying image acquisition conditions. We then employ Otsu’s thresholding method, which is an automated technique that determines optimal intensity thresholds by maximizing inter-class variance.

**Figure 7:**
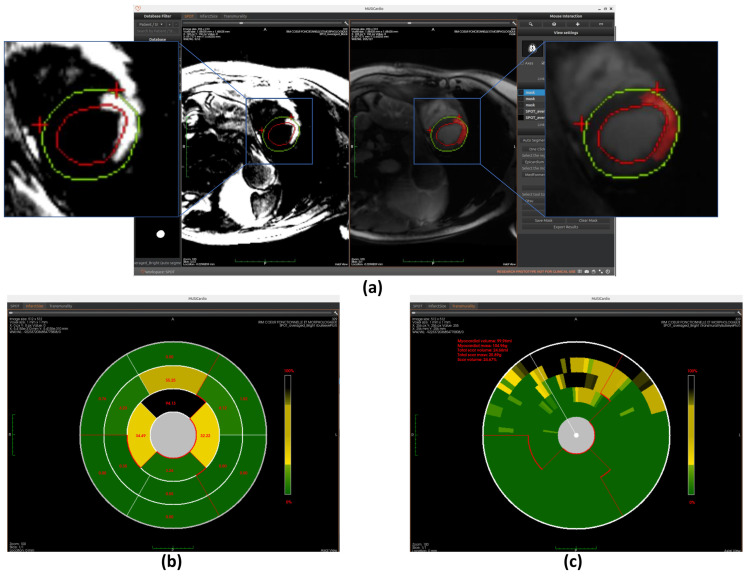
Overview of scar detection using the Otsu method. (**a**) Detected myocardial scar visualized on the BR-LGE image—highlighted as the red region between the left ventricular endocardium (red circle) and the epicardium (green circle), alongside comparison with the corresponding BL-LGE image. (**b**) Infarct size representation using an AHA 16-segment bullseye model. (**c**) Transmurality assessment using a bullseye plot with 100 equally spaced chords.

Our implementation first normalizes image intensities (0–255 range) before applying Otsu’s algorithm with three thresholds to classify pixels into distinct tissue categories (scar, gray zone, and healthy tissue). This provides an initial binary scar map that serves as input for subsequent refinement processes. The completely automated threshold selection requires no manual parameter adjustment, ensuring consistent performance across different cases (example of scar detection using Otsu is shown in Figure 7a).

#### 4.4.2. Scar Detection with 2D U-Net

**Figure 8:**
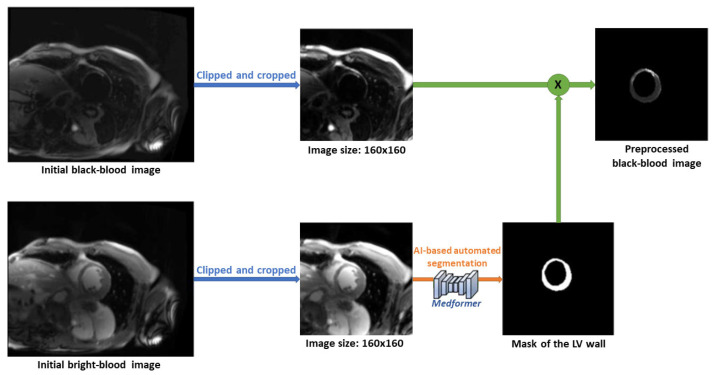
The pre-processing steps of automated scar detection method.

Our workspace also provides an alternative AI-based, fully automated scar detection method. The pre-processing steps are represented in the following Figure 8. First, the BR- and BL-LGE images are pre-processed separately. Both sets of images are center-cropped to 160 × 160 pixels, clipped to remove the brightest 2% of pixels, standardized using the dataset mean and standard deviation, and normalized to the range between 0 and 1. The preprocessed BR-LGE image is then used to automatically segment the LV wall using the MedFormer model. The resulting LV mask is multiplied with the corresponding pre-processed BL-LGE image to create a single input image that only contains scar information within the LV wall, which is then used for the neural network model.

**Figure 9:**
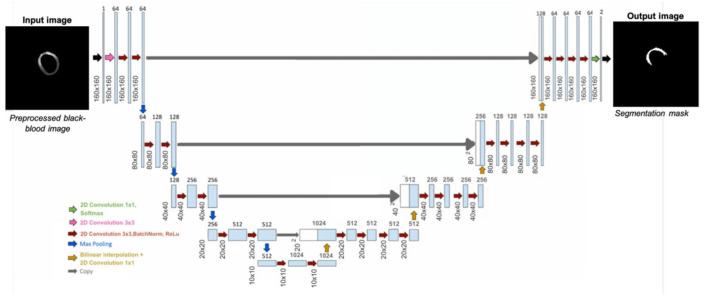
Framework of the 2D U-Net architecture used to detect potential myocardial scar [35].

This approach utilizes the U-Net neural network model (Figure 9) trained on data from 71 patients, including 6 healthy individuals. The dataset consists of 2D short-axis CMR images, with 3 to 18 slices per patient. After excluding apical and basal slices due to unreliable segmentation, a total of 595 co-registered BR- and BL-LGE images were used. Manual annotations of scar regions on BL-LGE images were provided by expert radiologists. The dataset was divided into training (70%), validation (10%), and test (15%) sets. A supervised learning strategy was applied, enabling the model to learn from labeled examples to accurately identify myocardial scars. On the test set, the trained model achieved an overall mean Dice score of 74.3%, confirming its reliability for automated scar detection.

**Figure 10:**
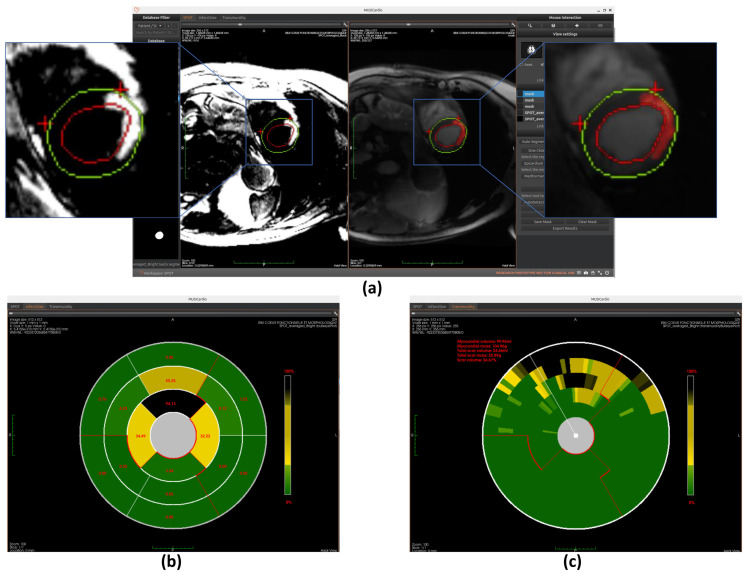
Overview of scar detection using the U-Net model. (**a**) Detected myocardial scar visualized on the BR-LGE image—highlighted as the red region between the left ventricular endocardium (red circle) and the epicardium (green circle), alongside comparison with the corresponding BL-LGE image. (**b**) Infarct size visualization using an AHA 16-segment bullseye model. (**c**) Transmurality evaluation presented with a bullseye plot composed of 100 equally spaced chords.

Similarly to the automated segmentation function, this model is implemented using the ONNX Runtime library to optimize inference performance within the workspace. The final detection results are displayed on BR-LGE images (shown in Figure 10a), enabling simultaneous comparison with scar visualization in corresponding BL-LGE images.

#### 4.4.3. Scar Detection with Region Growing

**Figure 11:**
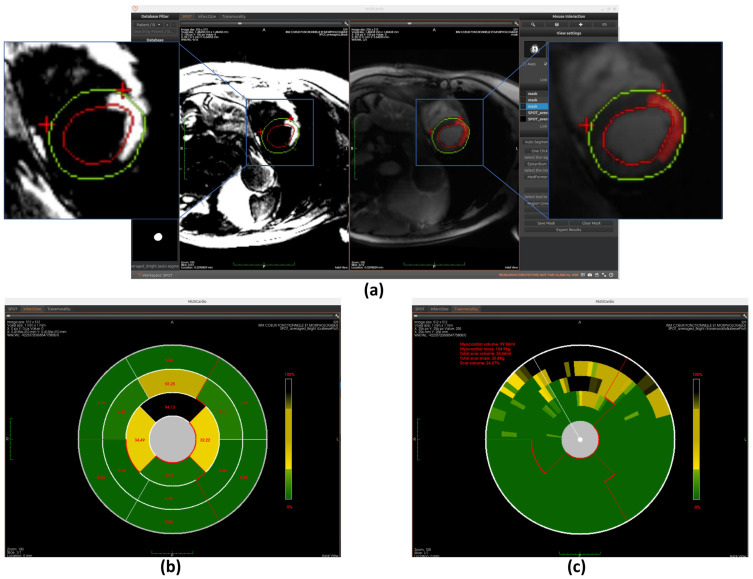
Overview of scar detection using the region growing method. (**a**) Detected myocardial scar visualized on the BR-LGE image—highlighted as the red region between the left ventricular endocardium (red circle) and the epicardium (green circle), alongside comparison with the corresponding BL-LGE image. (**b**) Infarct size visualization using an AHA 16-segment bullseye model. (**c**) Transmurality evaluation presented with a bullseye plot composed of 100 equally spaced chords.

The region growing algorithm provides a semi-automated approach for myocardial scar detection through iterative pixel aggregation. Beginning from a user-defined seed point, in our case, it is the gravity center, and the method expands the region by progressively incorporating adjacent pixels that meet predefined similarity criteria. The algorithm operates under spatial confinement within the epicardial boundary and intensity homogeneity relative to the evolving region mean as constraints. During each iteration, the 4-connected neighborhood of the current region is evaluated, with qualifying pixels added to a dynamically managed candidate list. The pixel exhibiting minimal intensity deviation from the current region mean is incorporated into the growing scar region, followed by immediate recalculation of the updated region statistics. This adaptive process continues until either the maximum allowed intensity deviation is exceeded or the entire myocardial area has been evaluated. The final binary output delineates the detected scar region while maintaining topological consistency with myocardial anatomy. (The detection result is shown in Figure 11a) This method complements fully automated detection approaches by allowing refinement of scar boundaries.

### 4.5. Myocardial Scar Quantification

The workspace’s final core function enables comprehensive quantification of detected scars, providing two distinct analytical models: infarct size quantification and transmurality quantification. These quantitative analyses are presented through intuitive bullseye plot visualizations that follow established cardiac segmentation standards. The technical implementation of each quantification method will be explained in detail in subsequent sections. Finally, the system compiles all quantitative findings into a structured clinical report formatted for diagnostic use.

#### 4.5.1. Scar Size Quantification

The first quantification model measures infarct size using the AHA 16-segment bullseye model. Cardiac MRI slices are divided into three anatomical regions (apex, mid-ventricle, and base) with each region further partitioned into 4–6 segments. The initial segmentation is guided by anatomical landmarks, as explained in Section 4.3. Specifically, for basal and mid-ventricular slices, the segmentation is achieved by bisecting the angle between the two predicted landmarks to define the midline, and subdividing the surrounding angle space into equal parts to generate additional points. For apical slices, direction vectors from each landmark to the image centroid are computed and reversed to define the remaining segment boundaries. This landmark-driven approach enables anatomically consistent and reproducible segmentation across different slices and subjects.

Once the segments are accurately divided, the model calculates the scar area as a percentage of the total area within each segment. This is done by measuring the number of pixels corresponding to the scar in each segment and dividing it by the total number of pixels in that segment. These percentage values are then mapped to the corresponding positions on the bullseye plot, visually representing the spatial distribution of myocardial scars, as shown in Figure 7b, Figure 10b, and Figure 11b.

#### 4.5.2. Scar Transmurality Quantification

The second quantification model evaluates myocardial scar transmurality using a 100-chords bullseye representation [4,36]. Each short-axis cardiac MRI slice is radially partitioned into 100 evenly spaced chords, drawn perpendicularly to a centerline positioned equidistant between the endocardial and epicardial contours, and extending from the endocardial to the epicardial border. Initial segmentation is guided by anatomical landmarks—particularly the insertion points between the right ventricle and the interventricular septum—which define the angular reference for chords location. These landmarks allow for the consistent alignment of the radial divisions across slices and subjects, ensuring accurate structural correspondence.

Once the segmentation is established, the model quantifies transmurality by assessing the proportion of scar tissue along each chord. For each chord, the ratio of scar thickness to the total myocardial wall thickness along the corresponding radial path is computed, reflecting the extent of myocardial wall affected. These transmurality values are then visualized in the bullseye plot, offering a high-resolution visualization of scar depth throughout the myocardium, as shown in Figure 7c, Figure 10c, and Figure 11c.

Concurrently, the workspace provides quantitative data outputs displayed adjacent to the bullseye plot, allowing clinicians to further analyze the results and integrate them into their diagnostic workflow.

#### 4.5.3. Report Generation

**Figure 12:**
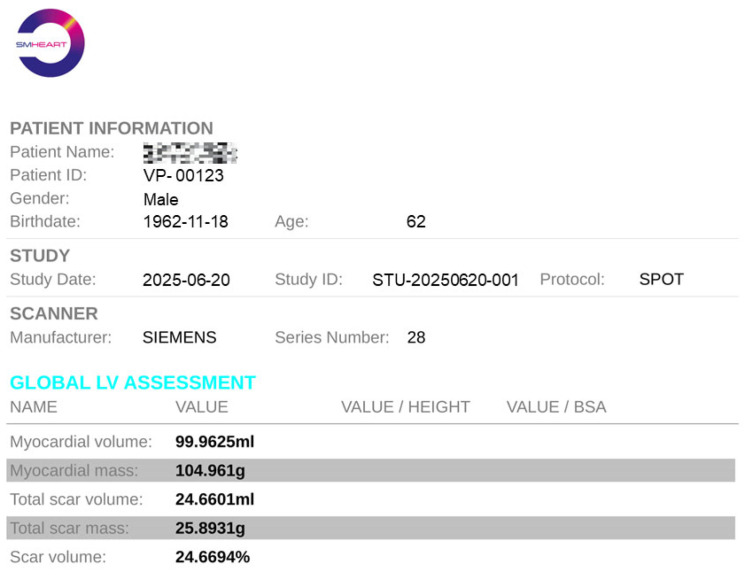
An example of quantitative analysis report with an anonymous patient and metric summary.

Upon completion of all quantitative analyses, the report generation option will become available. After selecting the file storage location, a clinical report for the current patient will be automatically generated. As illustrated in Figure 12, the report includes not only the patient’s basic information but also all calculated metrics such as myocardial volume, scar volume, and other relevant parameters. This standardized report can be directly utilized by clinicians or patients for clinical diagnostic purposes.

## 5. Conclusions

In this paper, we present SPOT-Cardio, an AI-powered imaging analysis toolbox designed for the automatic assessment of myocardial scars using both BR- and BL-LGE images acquired with the SPOT technique. Based on the various technical components detailed throughout the paper, SPOT-Cardio integrates AI models to fully automate essential clinical tasks: segmentation of the LV, detection and characterization of myocardial scars, landmark-based region localization, and quantification of key clinical metrics such as scar volume, extent, and transmurality. By streamlining the entire workflow, SPOT-Cardio greatly reduces the need for manual input, enhances reproducibility, and simplifies cardiac MRI analysis. Its user-friendly design supports one-click operation, making it easily deployable for routine clinical use.

Looking forward, this toolbox provides a strong foundation for further development. Its modular structure allows the integration of advanced cardiac tissue characterization techniques, such as T2 mapping [37] or other SPOT-based imaging modalities. These extensions will expand SPOT-Cardio’s diagnostic capabilities, enabling not only scar detection but also comprehensive myocardial tissue analysis, including fibrosis, inflammation, or edema evaluation. In clinical contexts, automated myocardial scar assessment provides valuable insights across a range of cardiovascular conditions. For example, in acute myocardial infarction, it enables quantification of infarct size and transmurality, supporting evaluation of functional recovery and guiding therapeutic decisions; in post-ablation patients, it allows assessment of fibrosis and helps predict the risk of arrhythmia recurrence; and in cardiomyopathies, automated scar analysis can reveal abnormal myocardial remodeling and assist in arrhythmic risk stratification.

In future work, we also plan to validate and refine SPOT-Cardio using independent clinical datasets. Clinicians will use the software in real-world settings, allowing us to collect their feedback and compare the automated results with assessments obtained using their current standard methods. This prospective validation will help evaluate the software’s impact on workflow efficiency and diagnostic accuracy.

Ultimately, SPOT-Cardio represents a step forward in intelligent cardiovascular imaging by bridging state-of-the-art image acquisition techniques with clinically actionable insights through the power of AI and automation.

## 6. Patents

All patents that have been granted and are relevant to our study can be seen in Table 2.

## Figures and Tables

**Table 1:** External libraries used in medInria, the core of MUSICardio.

Library	Description	Origin
DCMTK [21]	DICOM management	OFFIS e.V
dtk	Tools for modular software development	Inria
ITK	Scientific imaging management	Kitware
LCC LogDemons [22]	LCC Log Demons algorithms	Inria
QtDCM [23]	Qt widgets to handle DICOM images	Inria
RPI	Image registration algorithms	Inria
TTK	Tensor algorithms	Inria
VTK [24]	Image processing, 3D graphics, volume rendering, and visualization	Kitware
ONNX Runtime	Machine learning model inference acceleration	Microsoft

**Table 2:** List of related patents.

Ref OAK	Short Title	Filing Number	Priority Date	Countries
50348	Ti-Scout Black blood LGE	FR2203766	22 April 2022	FR, EP, US, CN
50349	Sequence Spot	FR2203782	22 April 2022	FR, EP, US, CN
50439	Post-processing Imaging	FR2300699	25 January 2023	FR, WO

## Data Availability

The data that support the findings of this study are not publicly available. Restrictions apply due to privacy and ethical considerations.

## Abbreviations

The following abbreviations are used in this manuscript:

AHA	American Heart Association
AI	Artificial Intelligence
BL-LGE	Black-blood
BR-LGE	Bright-blood
CMR	Cardiac Magnetic Resonance
CN	China
CNN	Convolutional Neural Network
CT	Computed Tomography
CVD	Cardiovascular Disease
EP	Europe
FCN	Fully Convolutional Network
FR	France
GUI	Graphical User Interface
I/O	Input/Output
LGE	Late Gadolinium Enhancement
LV	Left Ventricular
MRI	Magnetic Resonance Imaging
SD	Standard Deviation
SPOT	Scar-specific imaging with Preserved myOcardial visualizaTion
US	United States
